# P-868. Creation of a Novel OUD-Specific Antibiogram

**DOI:** 10.1093/ofid/ofaf695.1076

**Published:** 2026-01-11

**Authors:** Zachary Nichols, Matthew Pettengill, Nathaniel Hurwitz, Bryan Hess, Carolyn Kramer, Elizabeth Novick, John Zurlo, Rebecca Jaffe, Nikhil Seval

**Affiliations:** ChristianaCare, Philadelphia, PA; Department of Clinical Laboratories, Thomas Jefferson University Hospitals, Philadelphia, PA; Unaffiliated, Philadelphia, Pennsylvania; Sidney Kimmel Medical College at Thomas Jefferson University, Philadelphia, PA; Hospital of the University of Pennsylvania, Philadelphia, Pennsylvania; Thomas Jefferson University Hospital, Philadelphia, Pennsylvania; Jefferson University, Hershey, PA; Thomas Jefferson university, Philadelphia, Pennsylvania; Drexel University College of Medicine, Philadelphia, Pennsylvania

## Abstract

**Background:**

People who inject drugs are host to a clinically distinct microbial ecology given the population’s high pathogen burden and repeated, incomplete antibiotic exposures. Syndromic antibiograms have a potential utility in this population, and have not to our knowledge been used in patients with opioid use disorder (OUD) and may inform clinically relevant practice changes.

**Figure:**
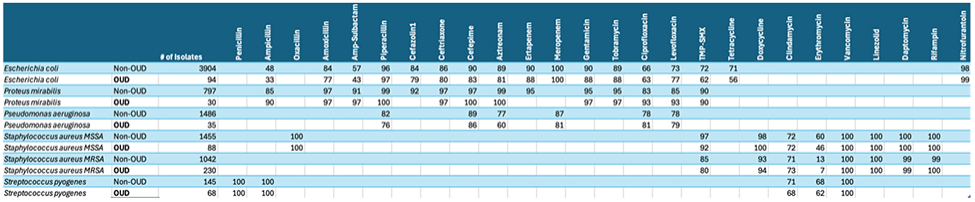
OUD Antibiogram Table The completed antibiogram table comparing the OUD population we identified with the general institutional population.

**Figure:**
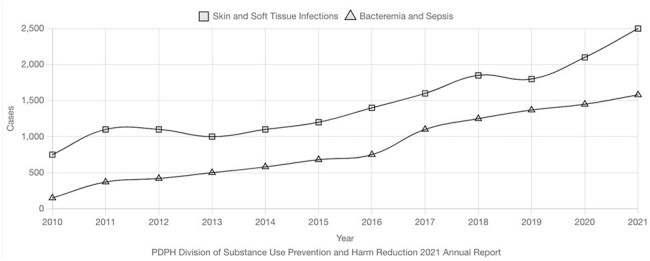
Hospitalizations for Substance Use-Associated Infections in Philadelphia, 2010-2021 Citywide hospitalization trends for skin and soft tissue infection and bacteremia in patients with substance use disorder show clear upward trajectories.

**Methods:**

We conducted a retrospective study of adults (≥18 years) admitted to Thomas Jefferson University Hospital or Jefferson Methodist Hospital during 2022-2023. Persons with OUD were identified by presence of OUD ICD diagnoses (F11.x ICD10 codes) and a positive fentanyl urine test, and positive cultures were included. Patients under 18 were excluded. Patient records were identified through EPIC and cross-referenced with Clinical Microbiology Laboratory data. We analyzed susceptibility patterns for common injection-related organisms (if ≥30 isolates present) and compared them with institutional antibiogram data after removing the OUD population.

**Figure:**
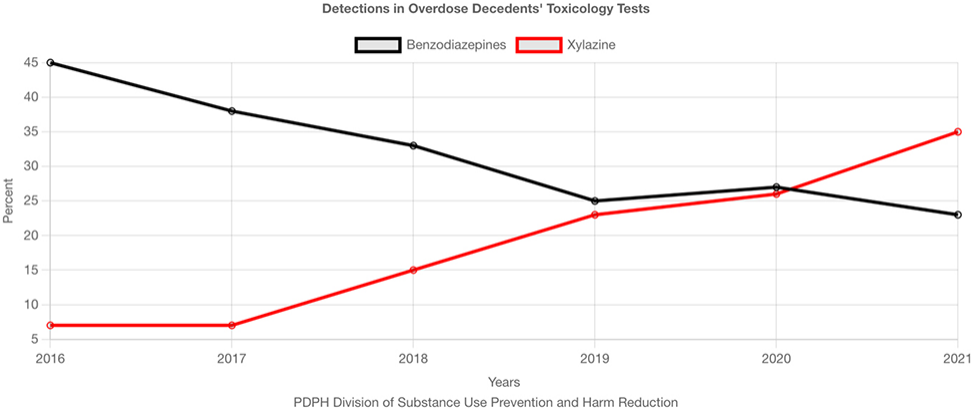
Detections in Overdose Decedents' Toxicology Tests The prevalence of benzodiazepines versus xylazine in post-mortem toxicology in Philadelphia, showing xylazine's growing market share.

**Results:**

1807 patients met OUD criteria over the two-year period. Susceptibility data was obtained for *Staphylococcus aureus*, *Streptococcus pyogenes*, *Pseudomonas aeruginosa*, *Proteus mirabilis*, *E. coli* for both the OUD group and total group, and an antibiogram was created as per institutional protocol. Cochran–Mantel–Haenszel testing showed a significant dependence (p=2.35e-8) between OUD and susceptibility when controlling for antibiotic-organism pairs. TMX-SMX susceptibility amongst MSSA isolates was 97% (non-OUD) versus 92% (OUD)(p=.012), and amongst MRSA isolates was 85% (non-OUD) versus 80% (OUD)(p=.087). Significantly increased resistance patterns were seen amongst *E. Coli* isolates in the non-OUD cohort versus OUD (ampicillin-sulbactam 57% vs 43%, p=.006; TMP-SMX 72% vs 62%, p=.012, ampicillin 48% vs 3%, p=.004). 32% of all *S. pyogenes* isolates for the given culture sites were from persons with OUD.

**Conclusion:**

A novel customized antibiogram that showed statistically and clinically significant differences in antibiotic susceptibility rates in the inpatient population with chart-identified OUD and identified valuable population level infection prevalence data.

**Disclosures:**

All Authors: No reported disclosures

